# 

**Published:** 1890-06

**Authors:** 


### BOOKS AND PAMPHLETS RECEIVED.

Cyclopedia of the Diseases of Children, Medical and Surgical. Edited by John M. Keating, M. D. Vol. iii. Illustrated. Philadelphia: J. B. Lippincott Co. 1890.

Electricity in the Diseases of Women. By G. Betton Massey, M. D., Physician to the Gynecological Department of Howard University, Late Electro-therapeutist to the Philadelphia Orthopedic Hospital, etc. Second edition; revised and enlarged. Philadelphia and London: F. A. Davis, Publisher. 1890.

New York State Board of Health. Ninth Annual Report. 1889. Transmitted to the legislature, Feb. 26, 1889. Albany: The Troy Press Co., Printers. 1889. From Hon. Leroy Andrus.

Stories of a Country Doctor. By Willis P. King, M. D., Member of the American Medical Association, Member and Ex-President of the Missouri State Medical Association, Assistant-Chief Surgeon of the Missouri Pacific Railway Co., Formerly Lecturer on Diseases of Women in the Medical Department of the Missouri State University, and Professor of Diseases of Women in the Medical Department, University of Kansas City, etc., etc. With illustrations by T. A. Fitzgerald. Kansas City, Mo.: Hudson-Kimberly Publishing Company. 1890.

Wood's Medical and Surgical Monographs. Vol. vi., No. 2. Contents: Insanity at the Puerperal, Climacteric and Lactational Periods, by W. Bevan Lewis, L. R. C. P. Treatment of Diseases of Women by Massage, by Dr. Robert Ziegenspeck, Munich. The Treatment of Internal Derangements of the Knee-Joint by Operation, by Herbert William Allingham, F. R. C. S. The Idiopathic Enlargement of the Heart, by Dr. Oscar Fraentzel, Berlin. May, 1890. New York: William Wood & Company, 56 and 58 Lafayette place. 1890.

Revue Internationale de Bibliographie Medicale, Pharmaceutique et Vétérinaire. Dirigée par Le Docteur Jules Rouvier. Paris Librairie Medicale Vve. Jacques Lechevalier, 23 Rue Racine.

Transactions of the Washington Obstetrical and Gynecological Society. Vol. ii. Oct. 7, 1887, to May 17, 1889. Press of Stettiner, Lambert & Co., 22, 24, 26 Reade street, New York.

How to Preserve Health. By Louis Barkan, M. D. American News Company. New York. 1890.

Report on Medical Education, Medical Colleges, and the Regulation of the Practice of Medicine in the United States and Canada. 1765–1890. Illinois State Board of Health. By John H. Rauch, M. D., Secretary. Springfield, Ill.: H. W. Rokker, Printer and Binder. 1890.

Forty-seventh Annual Report of the Managers of the State Lunatic Asylum at Utica, for the year ending Sept. 30, 1889. Transmitted to the Legislature, March, 1890. Albany: James B. Lyon, State Printer. 1890.

A Treatise on Orthopedic Surgery. By Edward H. Bradford, M. D., Surgeon to the Children's Hospital, Boston City Hospital, and Samaritan Hospital, Instructor in Clinical Surgery, Harvard Medical School; and Robert W. Lovett, M. D., Surgeon to the Samaritan Hospital, Assistant Out-patient Surgeon to the Children's Hospital, etc. Illustrated with 789 wood engravings. Specialties in the Practice of Medicine. New York: William Wood & Company. 1890.

A Discussion of the General Principles Involved in the Operation of the Removal of the Uterine Appendages. By Lawson Tait, F. R. C. S., Birmingham, Eng., President of the British Gynecological Society, etc. Reprint from the New York *Medical Journal* for Nov. 20, 1886.

Misplacements of the Uterus. History of Cases, Showing How in Many Instances they are Produced; the Accompanying Conditions; Microscopical Examinations. By Mary A. Dixon Jones, M. D., Brooklyn, N. Y. Reprint from the Pittsburgh *Medical Review*, October, 1889.

A Hitherto Undescribed Disease of the Ovaries. Endothelioma, Changing to Angeioma and Hematoma. By Mary A. Dixon Jones, M. D., Brooklyn. Reprint from the New York *Medical Journal* for September 28, 1889.

The Animal Suture, Its Place in Surgery. By Henry O. Marcy, A. M., M. D., LL. D., of Boston, U. S. A. Reprint from the Transactions of the American Association of Obstetricians and Gynecologists, September, 1889. Philadelphia: Wm. J. Dornan, Printer. 1889.

Exploratory Laparatomy. By Henry O. Marcy, A. M., M. D., LL. D., of Boston, Mass. Read in the section on surgery at the thirty-ninth annual meeting of the American Medical Association, May, 1888. Reprint from the *Journal of the American Medical Association*, January 26, 1889. Chicago: Printed at the office of the Association. 1889.

Twenty Consecutive Cases of Abdominal Section. By L. S. McMurtry, A. M., M. D., of Louisville, Ky. Reprint from the Transactions of the Southern Surgical and Gynecological Association, Nov. 1889. Philadelphia: Wm. J. Dornan, Printer. 1890.

Are the German Schweine-Seuche and the Swine Plague of the Government of the United States Identical Diseases? By Frank S. Billings. From the *American Naturalist*, April, 1890.

Evidence Showing that the Report of the Board of Inquiry Concerning Swine Disease was Fixed, and an Address on Original Research in Nebraska. By Frank S. Billings, Lincoln, Neb. State Journal Company, Printers. 1890.

Injuries of the Bladder during Laparatomy, Including a Report of Sixty-seven Cases. By A. Reeves Jackson, A. M., M. D. Reprint from the *Journal of the American Medical Association*, February 22, 1890. Chicago: Printed at the office of the Association. 1890.

A Lecture on Sexual Perversion, Satyriasis and Nymphomania. By G. Frank Lydston, M. D., Chicago. Reprint from Philadelphia *Medical and Surgical Reporter.*

Remarks on Hypertrophy and Atrophy of Tissue. By G. Frank Lydston, M. D., Chicago. New Orleans *Medical and Surgical Journal.*

The Local Treatment of Syphilitic Phenomena. By G. Frank Lydston, M. D., Chicago. Reprint from the Cincinnati *Lancet-Clinic.*

A Practical Splint for Inflammatory Conditions of Joints. By Charles F. Stillman, M. Sc., M. D. Reprint from *American Lancet*, March, 1890.

A Rational Brace for the Treatment of Caries of the Vertebræ. By Charles F. Stillman, M. D., M. Sc., of Chicago. Reprint from the Northwestern *Medical Journal.* 1890.

The Treatment of Torticollis. By Charles F. Stillman, M. Sc., M. D., of Chicago. Reprint from the *North American Practitioner.* March, 1890.

Large Doses of Iodide of Potassium. By Augustus A. Eshner, M. D., Resident Physician at the Philadelphia Hospital. From the *Medical and Surgical Reporter*, November 23, 1889.

The Blunt Curette in Uterine Hemorrhage. By Thomas W. Kay, M. D., Scranton, Pa. Reprint from the New York *Medical Journal* for November, 1889.

De La Mobilisation de Létrier. Par Le Docteur E. J. Moure. Communication faite au Congrés Internationale d'Otologie et de Laryngologie, de Paris, 1889. Paris. Octave Doin, Editeur 8, Place de L'Odéon. 1890.

Bericht über 50 Ovariotomien, ausgeführt von B. S. Schultze in Jena in der Jahren 1884,-85,-86 und '87. Inaugural Dissertation von Carl Purrucker-aus-Zeitz. Jena, Frommannsche Buchdruckerei. 1889.

Ueber Tetanus Puerperalis im Anschluss an Zwei Beobachtete Fälle˙ Inaugural Dissertation von Karl Witthauer. Jena, 1889. Druck von G. Neuenhahn.

Statistische Mittheilungen über Geburtshülfliche Operationen an der Frauenklinik zu Jena in den letzten 25 Jahren. Inaugural Dissertation von R. Hoffeman-aus-Kottbus. Jena, 1889. Druck von G. Neuenhahn.

Fifth Annual Report of the New York Cancer Hospital. 1889.

New York Post-Graduate School and Hospital, Eighth Annual Announcement. Sessions 1889-90.

Arguments Before the Assembly Committee on Codes, April 23, 1890, of Tracy Becker, Esq., Dr. R. A. Witthaus, and of Dr. Maurice J. Lewi, in Favor of the Enactment of Hon. R. P. Bush's Bill to Amend Section 306 of the Penal Code in Regard to Embalming Dead Bodies. Buffalo, N. Y. Printing House of James D. Warren's Sons. 1890.

Electrolysis in the Treatment of Stricture of the Rectum. By Robert Newman, M. D., of New York. Reprint *Journal American Medical Association*, May 17, 1890.

The Anniversary Address before the Medical Society of the State of New York. By Daniel Lewis, A. M., M. D., president. Reprint from the Transactions. 1890.

Pulmonary Consumption in the Light of Modern Research. By Stephen Smith Burt, M. D. From the *New York Medical Record*, April 12, 1890.

A History of Spectacles. By L. Webster Fox, M. D. From the *Medical and Surgical Reporter*, May 3, 1890.

The Sewer Gas Question. By E. S. McClellan, M. D., New York. Bartram's Press, 126 William street, New York.

Cornell University College of Agriculture. Bulletin of the Agricultural Experiment Station. XVI. March, 1890. Growing Corn for Fodder and Ensilage.

Abstract of Sanitary Reports. No. 16. Small-pox in Kentucky—Request for Protection by the Health Authorities of Tennessee. No. 17. Investigation of the Reported Endemic of Small-pox in Kentucky. No. 18. Louisiana– Annual Proclamation of Quarantine. No. 19. A Case of Leprosy in Indiana. Nos. 20, 21 and 22.

State Board of Health of New York. Monthly Bulletins for March and April, 1890.

State Board of Health of Tennessee. Monthly Bulletin, May 20, 1890.

Newport, R. I., Reports of Deaths and Contagious Diseases for April and May, 1890.

Health in Michigan, April and May, 1890.

---

### Literary and Other Notes.

THE WILLIAM F. JENKS MEMORIAL PRIZE.—The second Triennial Prize of four hundred and fifty dollars, under the deed of trust of Mrs. William F. Jenks, will be awarded to the author of the best essay on The Symptomatology and Treatment of the Nervous Disorders Following the Acute Infectious Diseases of Infancy and Childhood.

The conditions annexed by the founder of this prize are, that the "prize or award must always be for some subject connected with Obstetrics, or the Diseases of Women, or the Diseases of Children;" and that "the Trustees, under this deed for the time being, can, in their discretion, publish the successful essay, or any paper written upon any subject for which they may offer a reward, provided the income in their hands may, in their judgment, be sufficient for that purpose, and the essay or paper be considered by them worthy of publication. If published, the distribution of said essay shall be entirely under the control of said trustees. In case they do not publish the said essay or paper, it shall be the property of the College of Physicians of Philadelphia."

The prize is open for competition to the whole world, but the essay must be the production of a single person.

The essay, which must be written in the English language, or if in a foreign language, accompanied by an English translation, should be sent to the College of Physicians of Philadelphia, Pennsylvania, U. S. A., before January 1, 1892, addressed to Louis Starr, M. D., Chairman of the William F. Jenks' Prize Committee.

Each essay must be distinguished by a motto, and accompanied by a sealed envelope bearing the same motto and containing the name and address of the writer. No envelope will be opened except that which contains the successful essay.

The committee will return the unsuccessful essays if reclaimed by their respective writers, or their agents, within one year.

The committee reserves the right not to make an award if no essay submitted is considered worthy of the prize.

---

THE MEDICAL PROFESSION OF THE STATES OF NEW YORK, OHIO, ILLINOIS, INDIANA, AND IOWA. The undersigned requests the members of the medical profession in the above-named States to forward, at their earliest convenience, the following points :

Name in full . . . . . . . . . . . . . . . . . . . . . . School of Graduation and Year . . . . . . . . . . . . . . . . Post-office address . . . . . . . . . . . . . . . . . . . .State. . . . . . . . . . . . . . . .

This will be used in the pages of the *Medical Register, Directory and Intelligencer*, Dr. William B. Atkinson, editor. A copy of the book, printed on good paper, nicely bound, will be forwarded to each physician (whose name appears in its pages) without charge.

The matter in preparation for it is of such value to the profession that everyone who receives a copy will be sure to keep it at hand for constant reference. Its list of National and local medical organizations and post-office addresses of physicians will be complete to date of issue, besides other valuable information. Address, George Keil, publisher, 1214 Filbert street, Philadelphia.

---

INTUBATION OF THE LARYNX. The thesis of Dr. Arthur G. Root, class of '90, Albany Medical College, upon Intubation of the Larynx, received the highest prize as an Inaugural Thesis, and appears in the *Medical Bulletin* for May. It gives a complete *résumé* of the history of the operation and the improvements introduced by Dr. O'Dwyer, of New York, upon the plan of Bouchut. The instruments, as well as the details of the operation, are carefully described, with explicit directions for managing the case before, during, and after the operation. The difficulties of the method disappear upon gaining the requisite manual dexterity, and every practical physician should be able to recognize the indications for intubation and be prepared to perform this little operation in order to save children from suffocation. It is no longer an experiment, but is used by the leading men in the profession, and has been performed nearly 3,000 times in this country. The notes of a number of clinical cases accompany this very interesting and timely article.

---

An Epitome of Examination of Recruits. By Charles R. Greenleaf, Major and Surgeon, U. S. A. This epitome has received the approval of the Secretary of War, and is the official standard for the physical examination of recruits for the U. S. Army, and for admission to the Military Academy at West Point, and to the Medical Corps, U. S. A.

The following extract from a note in the official register of the U. S. Military Academy, West Point, is equally applicable to candidates for admission to the Medical Corps, U. S. Army.

> It is suggested to all candidates for admission to the Military Academy that, before leaving their place of residence for West Point, they should cause themselves to be thoroughly examined by a competent physician, and by a teacher or instructor in good standing. By such an examination any serious physical disqualification or deficiency in mental preparation would be revealed and the candidate probably spared the expense and trouble of a useless journey and the mortification of rejection.

Price: 75 cents, postage prepaid. For sale by Wm. Ballantyne & Sons, 428 Seventh street, N. W., Washington, D. C.

---

Our esteemed contemporary, the Maryland *Medical Journal*, begins its twenty-third volume with improvements and changes which add much to its appearance and usefulness. Among these are a new cover, colored blue granite, a table of contents on the first page which can be bound in with the reading matter, and new, clean type. The *Journal* anticipates publishing during the summer months a series of short practical articles along with the usual original contributions. Special subjects will be treated by specialists, and regular letters from London and Paris, with occasional ones from other medical centers, are among the promised features of the future. We wish the *Journal* hearty success for the progressive spirit which it manifests in trying to please its patrons and elevate medical journalism.

---

Dr. J. D. Jackson, in the December number of the Omaha *Clinic*, gives his experience in the treatment of diphtheria by the iodide of sodium. He has treated fourteen cases, with one death. These cases were of the worst kind, and after giving the iodide of sodium for thirty-six hours the membranes disappeared from the throat and no bad symptoms followed. The iodide of sodium was given in 8–10 grain doses every hour for six hours, then every two or three hours. The author has never discovered any bad effects from its use.

---

ANY one interested in the sick benefit, funeral-aid, and death beneficiary associations of the United States can help make the statistics of their organizations for the forthcoming census more complete and disseminate the knowledge of the good work they are doing by sending the names of such societies as they may know of, and the addresses of their principal officers, to Mr. Charles A. Jenney, special agent of the eleventh census, 58 William street, New York City.

---

THE editorial article of the May issue of *The Dietetic Gazette* was prepared by J. Lewis Smith, M. D., Clinical Professor of Diseases of Children, in Bellevue Hospital Medical College. With the June number will begin an extended article on The Care and Feeding of Infants, with remarks on the Great Mortality of Infants in the Summer Months, and mode of preventing it.

---

DR. WM. T. BELFIELD, 612 Opera House building, Chicago, Ill., U. S. A., respectfully solicits information concerning unpublished cases of operations upon the prostate, especially for the relief of the so-called hypertrophy of the organ.

---

A CHECK TO CHRISTIAN SCIENCE.—Two "Christian Scientists" of Jamestown, N. Y., treated a patient who was suffering from cancer. She died, and a coroner's jury rendered the following verdict: "Mrs. Barrows came to her death from cancer of the breast on the 8th of May. We believe that contributory to this death was the culpable negligence of Mrs. M. J. Smith and Mrs. E. G. Lovejoy, who were advised of the fatal nature of the malady with which deceased was suffering, and failed to resort to or advise treatment by any of the methods known to medical science. We further believe that W. A. Barrows was also negligent of his duty in not securing medical treatment for his wife when there was reason for believing that she was in need of such treatment."—*Medical Record, May 31, 1890.*

